# The threat of a non-native oligochaete species in Iran's freshwater: assessment of the diversity and origin of *Eiseniella tetraedra* (Savigny, 1826) and its response to climate change

**DOI:** 10.1242/bio.060180

**Published:** 2023-12-29

**Authors:** Maryam Bagheri, Maryam Azimi, Hadi Khoshnamvand, Asghar Abdoli, Faraham Ahmadzadeh

**Affiliations:** Department of Biodiversity and Ecosystem Management, Environmental Sciences Research Institute, Shahid Beheshti University, G.C., Evin, Tehran 1983963113, Iran

**Keywords:** *Eiseniella tetraedra*, Non-native species, Genetic origin, SDMs

## Abstract

Oligochaetes are the most abundant benthic taxa in aquatic ecosystems that play an important role in food webs. The present study aims to assess the diversity and origin of *Eiseniella tetraedra* as a non-native species in the Lar National Park of Iran and also its response to current and future climate change. We obtained the specimen from rivers and sequenced the mitochondrial gene Cytochrome Oxidase subunit I (COI) and combined them with 117 sequences from the Jajroud and Karaj rivers in Iran and native regions from GenBank (NCBI). We also ran Species Distribution Modelings (SDMs) using an ensemble model approach that was estimated according to two shared socio-economic pathways (SSPs): 126 and 585 of the MRI-ESM2 based on CMIP6. According to the results, all the samples examined in the current study originated from Spanish rivers, and no unique haplotype was found in the Lar National Park. Moreover, the results also show high haplotype diversity that can positively affect the success of this non-native species in different freshwater. Also, the results of SDMs depict that climate change would remarkably affect the distribution of *E. tetraedra* and it verifies the invasion power of *E. tetraedra* in Iran's freshwater ecosystems over time.

## Teaser

**Summary:** A non-native dominant oligochaete species is a silent threat to the freshwater ecosystems of Iran.

## INTRODUCTION

Oligochaete species belonging to annelid worms occur in marine and terrestrial ecosystems. About one third of the almost 5000 known Oligochaete species is aquatic and semiaquatic (Suriani et al., 2007; Krieger and Stearns, 2010). With some exceptions, these groups of oligochaetes are small in size, ranging from 1 mm to a few cm in length (Martin et al., 2008). Regarding the idea that earthworms have a limited capacity to autonomously disperse (Sakai et al., 2001), it has been thought that the cosmopolitan dissemination of some oligochaetes (about 110 species) will often be due to human activities or animal-mediated transportation (Costa et al., 2013).

The occurrence of introduced species may cause ecological changes in the ecosystem if they can adapt to new habitats, leading to potential negative interaction with the native species (Gozlan and Newton, 2009; Davis et al., 2011). Several researches have demonstrated that introduced Oligochaete worms have remarkable impacts on the ecosystem. Once introduced, earthworms become invasive, and they can cause changes in microorganisms’ fauna of soil, competition with native species and loss of biodiversity resulting in economic losses and detrimental effects on habitats (Eisenhouer et al., 2019; Blouin et al., 2013; Craven et al., 2017). Furthermore, management options for controlling invasive species are generally troublesome and presumably threaten native species. Hence, clarifying the population structure of exotic species is essential to decrease their harmful effects on the ecosystem.

Parallel to the worldwide status, Iranian inland waters are exposed to habitat degradation and species introductions (either intentionally (e.g. for soil remediation or commercial applications) or inadvertently (e.g. in soil associated with horticultural and agricultural products) likely decrease endemic populations (Abdoli et al., 2022).

Untill now, 35 species of Oligochaetes have been identified from different regions of Iran (Ezzatpanah et al., 2010; Mirmonsef et al., 2011; Jabłońska and Pešić, 2014; Nazarhaghighi et al., 2014; Sanchooli et al., 2019; Latif et al., 2020). Some molecular surveys have been conducted to clarify whether these species are native or exotic (Latif et al., 2018; Bozorgi et al., 2019). Recently, Javidkar et al. (2020) reported a non-native Oligochaete *Eiseniella tetraedra* (Savigny, 1826) from two protected rivers in the southern Alborz Mountains. They deduced European origin for Iranian populations that were transported to this country by anthropogenic activities.

*Eiseniella tetraedra* is a fascinatingly specific occupant of waterlogged environments, specifically found along the shores of lakes and rivers and thrives in muddy habitats (Terhivuo, 1988; Schwert and Dance, 1979). This species is typically slow moving and sedentary but can exhibit wriggling motions to swim when disturbed. According to Terhivuo et al. (1994), in northern Europe, *Eiseniella tetraedra* exhibits a tetraploid genetic structure and parthenogenesis. Furthermore, in a study conducted by Mirmonsef et al. (2011), two distinct populations of this species were discovered in Tehran. One population was exclusively found alongside water courses, while the other population inhabited wet leaf litter. These populations exhibited variations in terms of size and coloration, despite belonging to the same species. Population genetic structure is the distribution of genotypes in space and time and is determined by both historical and current evolutionary processes (Hewitt and Butlin, 1997). The previous study (Javidkar et al., 2020) reported genetic variation in *E. tetraedra* populations in Iran that may be related to the successful establishment and colonization of the species in new habitats. However, the origin and distribution paths have not fully been investigated. For example, whether current populations are derived from a single introduction or are the result of several successive waves.

Besides molecular analysis, species distribution models (SDMs) can act as a useful tool to predict the distribution of species according to climate change. In fact, SDMs significantly relate species presence points with climate and topographical data, unveiling species-to-environment connections that are responsible for shaping species distribution patterns (Ashrafzadeh et al., 2022). However, for non-native species, it has been firmly documented that the SDMs approach could be a valuable proactive tool to distinguish invasion potential (Guisan and Thuiller, 2005; Reshetnikov and Ficetola, 2011; Gallien et al., 2012; Banha et al., 2017; Godefroid et al., 2020; Nunez-Mir et al., 2019).

The objective of this study is to combine molecular data and SDMs method to investigate the origin and differentiation of *E. tetraedra* populations from two protected rivers (Jajroud and Karaj) and one national park (Lar) located in the Southern Alborz Mountains. The study also aims to predict the potential distribution of *E. tetradra* based on climate change scenarios to determine its invasion potential.

## RESULTS

### Phylogenetic analyses and haplotype network

The datasets, with 528 bp length included partial sequences of Cytochrome c Oxidase subunit I (COI) to investigate the position of individuals belonging to the *E. tetraedra* in a phylogeny tree was constructed. The tree based on 140 sequences examined in this research contains the Lar National Park (23 sequences), the Jajroud (40 sequences), and the Karaj River (40 sequences), and 37 sequences from the native distribution area of the species were drawn.

The constructed intraspecies phylogenetic trees based on COI showed similar topologies for both ML and BI trees and revealed well-supported monophyletic lineages (only the BI tree shown, Fig. 2). In association with the outgroup, the *Hermodice carunculata* clustered as sister monophyletic lineages.

Based on the phylogenetic tree, the *E. tetradra* group was considered a well-supported monophyletic group as expected and the Iranian samples were well placed together with other samples of the population of origin. (Fig. 2).

The phylogenetic trees depict that the *E. tetraedra* group is well separated with high support values. The samples of this study belong to the Spanish catchment clade, which indicates the origin of the individuals studied in Iran.

The parsimony haplotype NETWORK for 140 *E. tetraedra* specimens (103 specimens from Iran and 37 specimens from the native distribution area) showed separate haplogroups and different haplotypes for COI by recognizable mutations (Fig. 3). It showed five haplogroups and 40 haplotypes for 140 *E. tetraedra* from the distribution area. Also, according to the results, four haplotypes were verified for the National Park as they were shared with the Jajroud and Karaj, and the Lar National Park region did not have a specific haplotype.

### Genetic diversity and demographic analysis

Demographic analyses for four localities from all regions were considered. The result of molecular diversity indices depicts the number of haplotypes (H), haplotype diversity (h), number of polymorphic sites (s), and nucleotide diversity (π) based on COI in Table 1.

**Table 1.**
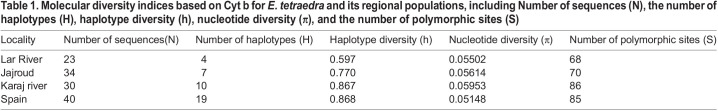
Molecular diversity indices based on Cyt b for *E. tetraedra* and its regional populations, including Number of sequences (N), the number of haplotypes (H), haplotype diversity (h), nucleotide diversity (π), and the number of polymorphic sites (S)

Tajima's D and Fu's fs analysis were not significant for each population (the Lar National Park population *P*<0.88, the Jajroud population *P*<0.98, the Karaj River population *P*<0.89, and Spanish population *P*<0.64) (Table 2). In addition, The MMD diagrams for all populations indicated a multimodal pattern (additional file: Fig. S1).

**Table 2.**
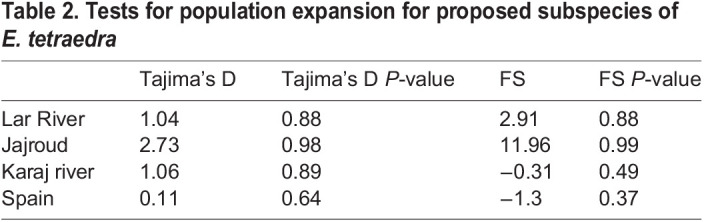
Tests for population expansion for proposed subspecies of *E. tetraedra*

### Modeling the present and future distribution

In all pattern predictions of models for *E. tetraedra* AUC (0.90-1.00), TSS (0.70-0.89), and KAPPA (0.78-0.92) were good to excellent predictive capacity. Also, the best-performing models for *E. tetraedra* were MaxEnt, GLM, ANN, and RF with AUC, TSS, and KAPPA >0.80 (Table S2).

Among variables BIO 1 (34.3), BIO 9 (24.8), BIO 14 (16.6), and Footprint (10.7) were the greatest contribution to model performance (Table 3). Likewise, temperature annual range (3.2%), precipitation of wettest month (3.1%), slope (2.8%), precipitation of wettest quarter (2.4) and mean temperature of wettest quarter (2.1%) were contributed to the model performance.

**Table 3.**
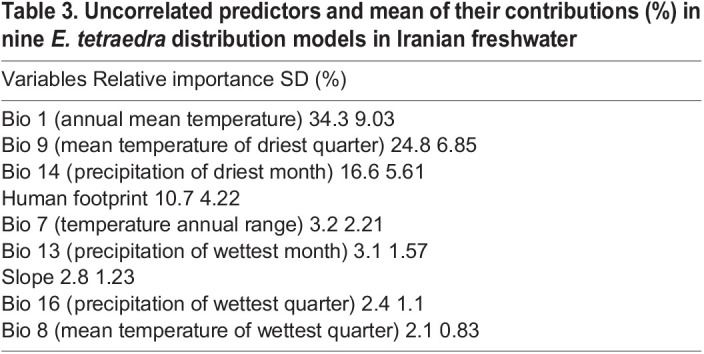
Uncorrelated predictors and mean of their contributions (%) in nine *E. tetraedra* distribution models in Iranian freshwater

According to the future climate change scenarios (SSP126 and 585) in our models projected the increase of suitable areas under all climate scenarios over time and suitable habitats will sharply increase over time across most of the *E. tetraedra* range (Fig. 4), as it tends to shift a wide range in the Western and almost the Southern of Iran.

## DISCUSSION

This study clearly reports the role of genetic data in the identification of the origin of *E. tetraedra* a non-native species in Lar River freshwater ecosystem. In fact, we investigated the origin of the introduction of *E. tetraedra* in Iran and tried to explain how the suitable habitat will change using the SDMs approach.

The beginning of studies on aquatic Oligochaeta back in 1920 (Stephenson, 1920) and after about 100 years the Iranian fauna of aquatic Oligochaeta is inadequately known and limited to just a few studies (Egglishaw, 1980; Ahmadi et al., 2012; Javidkar et al., 2020). Based on previous studies, 20 Oligochaeta species had been verified in Iran up to 2015 (Jabłońska and Pešić, 2014). Considering the area, mountainous landscapes, geographical features, and specific hydrological characteristics of Iran, it seems that there will be an increase in the number of these species in the future.

Latif et al. (2009) identified *E. tetraedra* based on morphology as a non-native species with European and Palearctic origin from the Haraz and Chalus rivers in Iran. Then Javidkar et al. (2020) reported the first molecular attempts to discover the aquatic oligochaetes in Iran and they confirmed the non-native species by combining samples from the Jajroud and Karaj with sequences from NCBI from studies elsewhere in the world for the species.

Until this study and Javidkar et al. (2020), the name of the species has not been listed in the native aquatic oligochaetes of Iran and our results reported *E. tetraedra* as a non-native species in the Lar National Park freshwater ecosystem. In line with our results, a number of researchers have reported *E. tetraedra* as a non-native species in other regions (Brinkhurst, 1960; Wood and James, 1993; Martinsson et al., 2015; de Sosa et al., 2017; Kim et al., 2017; Javidkar et al., 2020).

### Haplotype and genetic diversity

The results of the haplotype network clearly showed that the samples of the Lar National Park, the Jajroud, and Karaj rivers did not have a specific haplotype, and haplotypes of the current study are shared with the Jajroud and Karaj rivers. To explain this phenomenon three hypotheses can be suggested: (a) *E. tetraedra* was independently introduced into all three habitats in Iran; (b) initially, *E. tetraedra* was introduced in the Lar National Park, and then transferred to the Jajroud and Karaj river, and its diversity and abundance decreased over time in the Lar National Park; considering the Karaj river has the highest haplotype diversity and specific haplotype and also from the Karaj river to the Jajroud and Lar National Park, the haplotype diversity decreases; the third hypothesis (c) proposes *E. tetraedra* has initially introduced the Karaj river and was transferred to the Jajroud and then to the Lar National Park. However, based on the evidence and results, the third hypothesis is stronger. As well as, according to studies, altitude is one of the important limiting factors of distribution for the species as the abundance and diversity of Oligochaeta decrease with increasing altitude (Salomé et al., 2011). Considering that the Lar National Park is located at an altitude of about 3000 meters, it seems that the species was not native to the region and accidentally transferred to the area.

The molecular diversity indices depicted that the haplotype and genetic diversity within the species were almost high (Table 1). Also, according to Table 1, the π of *E. tetraedra* in the three Iranian populations were 0.05502e0.05953 and in the Spanish population was 0.05148 that it showed almost no different genetic diversity in all populations and the Iranian population has not low genetic diversity than the Spanish population. Therefore, not having low genetic diversity compared to the origin population, can express the invasivation of the species in the introduced areas. Xu et al. (2001) mentioned that for non-native species genetic diversity is necessary to adapt to new habitats and maintain new population sizes. In addition, having high haplotype diversity is one of the most important features affecting the success of invasivation of the species (Kolbe et al., 2004). The result (Table 1) showed that *E. tetraedra* haplotype diversity in Iran's freshwater is increasing and it will have invasive success in Iran's freshwaters.

### Species distribution modeling (present–future)

Our study showed the impact of climate change on the distribution range of non-native *E. tetrahedra* in Iran's freshwater ecosystems. Carosi et al. (2019) believed that species can experience four reactions under climate-change effects (i.e. expansion, reduction, both, or stability) in their habitats.

The current map for *E. tetrahedra* clearly shows the suitable distribution for the species, which could occur in a wider distribution range especially in some regions out of the recorded areas (Fig. 4). Based on the outcome future maps of climate change modeling under SSPs scenarios, it will be predicted that climate change would significantly affect the distribution of *E. tetrahedra* as maps showed a sharp tendency to expand over time in its distribution areas (Fig. 4). In connection with our results, Mamun et al. (2018) predicted future climate-change effects on an invasive alien species *Micropterus salmoides* in the Korean peninsula for 2050 and 2100. According to their results, the potentially suitable habitats for *M. salmoides* are most likely to increase by 2050 and 2100.

Moreover, regarding the output of the modeling, it seems where human population density is high, these areas are probably more affected by the species in the future. This may be due to the high human activities, travel, and trade in these areas.

The temperature increase is an effective factor in the expansion of *E. tetrahedra* in Iran's freshwater ecosystems as expected climate change would benefit the species. In fact, temperature and precipitation played the most important role in model predictions. Based on the studies, *E. tetraedra* is expanding in most regions of the world and usually prefers humid habitats (Latif et al., 2009; Ezzatpanah et al., 2010; Mirmonsef et al., 2011; Yousefi et al., 2009). Therefore, it may be possible to justify their distribution in the humid regions of the country, including the northern and southern regions. Hong et al. (2022) with SDMs tools mentioned temperature as the main reason for the expansion of range shifts in two invasive alien species under future climate-change scenarios.

However, the results of SDMs explicitly illustrated the invasion power of *E. tetrahedra* in Iran's freshwater ecosystems over time. It is mentioned that with expansions of alien species the vulnerability of native species will probably be more significant (Hansen et al., 2017; Abdoli et al., 2022; Kim et al., 2022) and it results in lowering the species diversity and degrading the sustainability of native freshwater species (Shi et al., 2010).

One of the main drivers of worldwide biodiversity loss is biotic exchange in ecosystems by invasive species (Butchart et al., 2010). Although we did not appraise the effects of *E. tetraedra* as a non-native on other non-oligochaete species, studies e.g. Migge-Kleian et al. (2006) and Ziemba et al. (2016) have shown the negative effects of non-native earthworms across trophic levels. According to the evidence in the present study and the identification of the success of Oligochaetes species in terms of being invasive in the river systems of Iran, it is assumed that freshwater ecosystems may be quite vulnerable to Oligochaetes of Western Palearctic origin and taking into account the negative consequences on native species, careful management strategies and regulations can help to mitigate these risks. It is essential that governments and individuals alike take a proactive approach to preventing the spread of invasive species and work to protect native ecosystems (Leprieur et al., 2008). Additionally, quarantine policies must be strictly enforced to help ensure that no potentially damaging organisms are imported into new environments.

*E. tetraedra* as an Oligochaete species is an interesting example of a non-native species in Iran's freshwater*.* Integrating the molecular data and SDMs approaches allowed us to unveil the successful biological invasion of *E. tetraedra* in Iran's freshwater. Our results provided the origin of specimens of this study and supplied a novel approach to assessing the biological invasion of *E. tetraedra* under climate change. Although the current study presented evidence for the invasion *E. tetraedra*, the information can help establish strategic and priority area data for ecosystem conservation.

## MATERIAL AND METHODS

### Taxon sampling and laboratory procedures

**Fig. 1.**
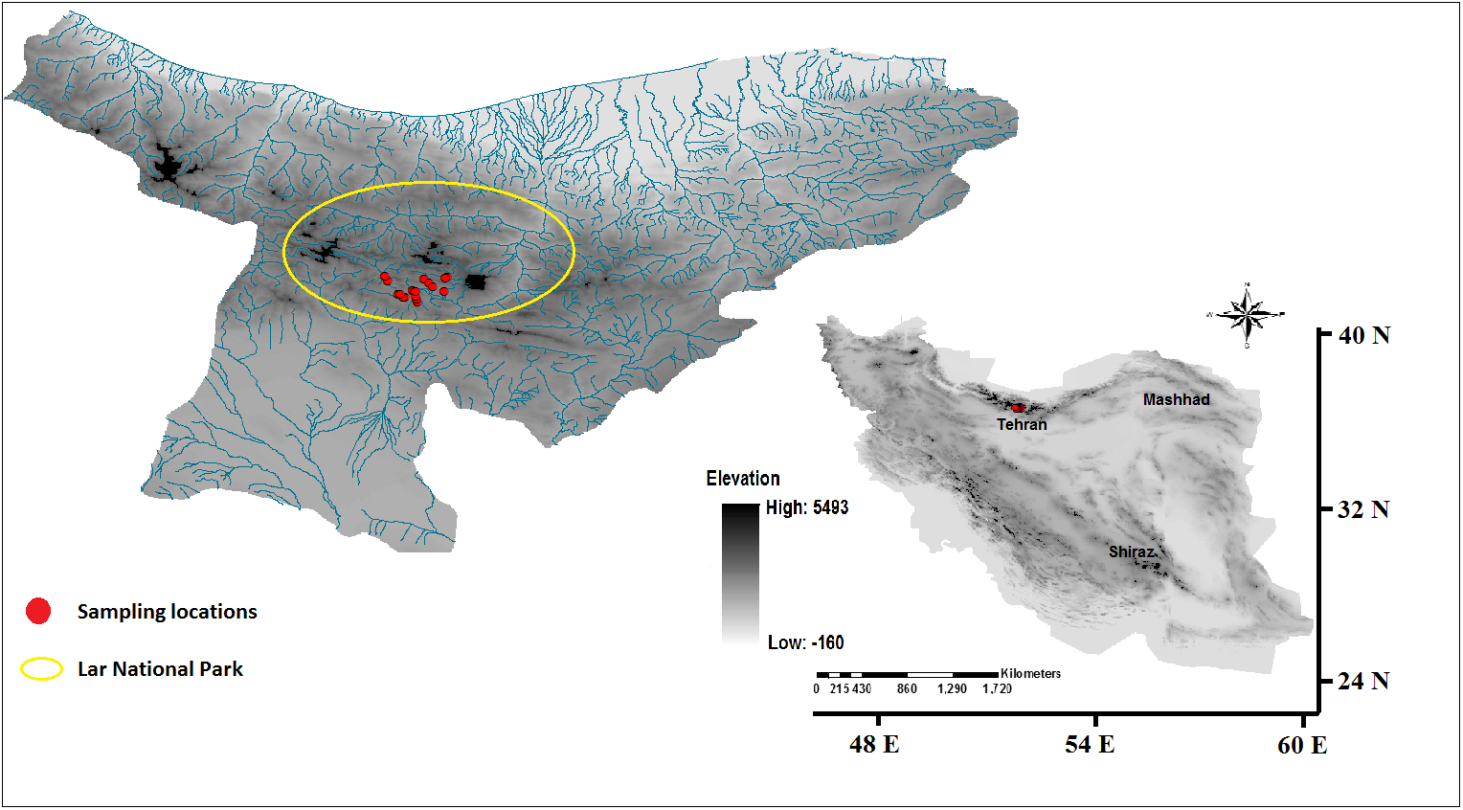
**Locations of sampling stations *E. tetraedra* in current study.** The location was made using the Digital Elevation Model (DEM) in ArcGis version 10.7.

In the current study, a total of 23 specimens of *E. tetradra* from 12 stations were collected from the rivers of the Lar National Park in Iran (Fig. 1). A small piece of the end of the body was dissected for each specimen then all tissues for DNA extraction were preserved in 96% ethanol and at −20°C. Locality and collection data for *E. tetraedra* and the sequences used in NCBI are explained in an additional file: Table S1.

**Fig. 2.**
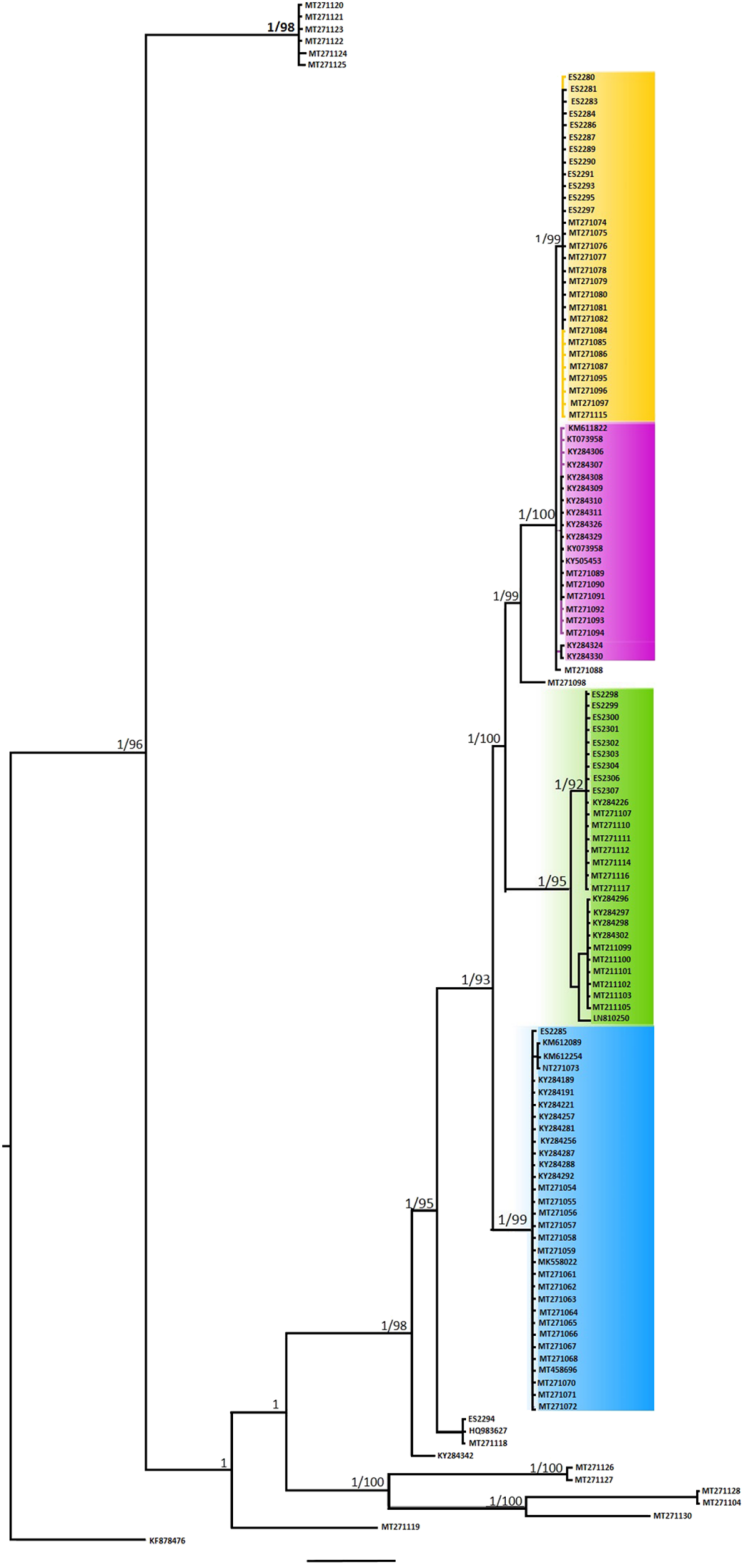
**Phylogenetic tree reconstructed for *E. tetraedra* based on the COI plus additional sequences accessed through GenBank and out group.** For each node, nodal supports indicate BI posterior probabilities (top) and ML bootstrap support (in percent, base). The scale bar represents 0.07 substitutions per nucleotide position.

**Fig. 3.**
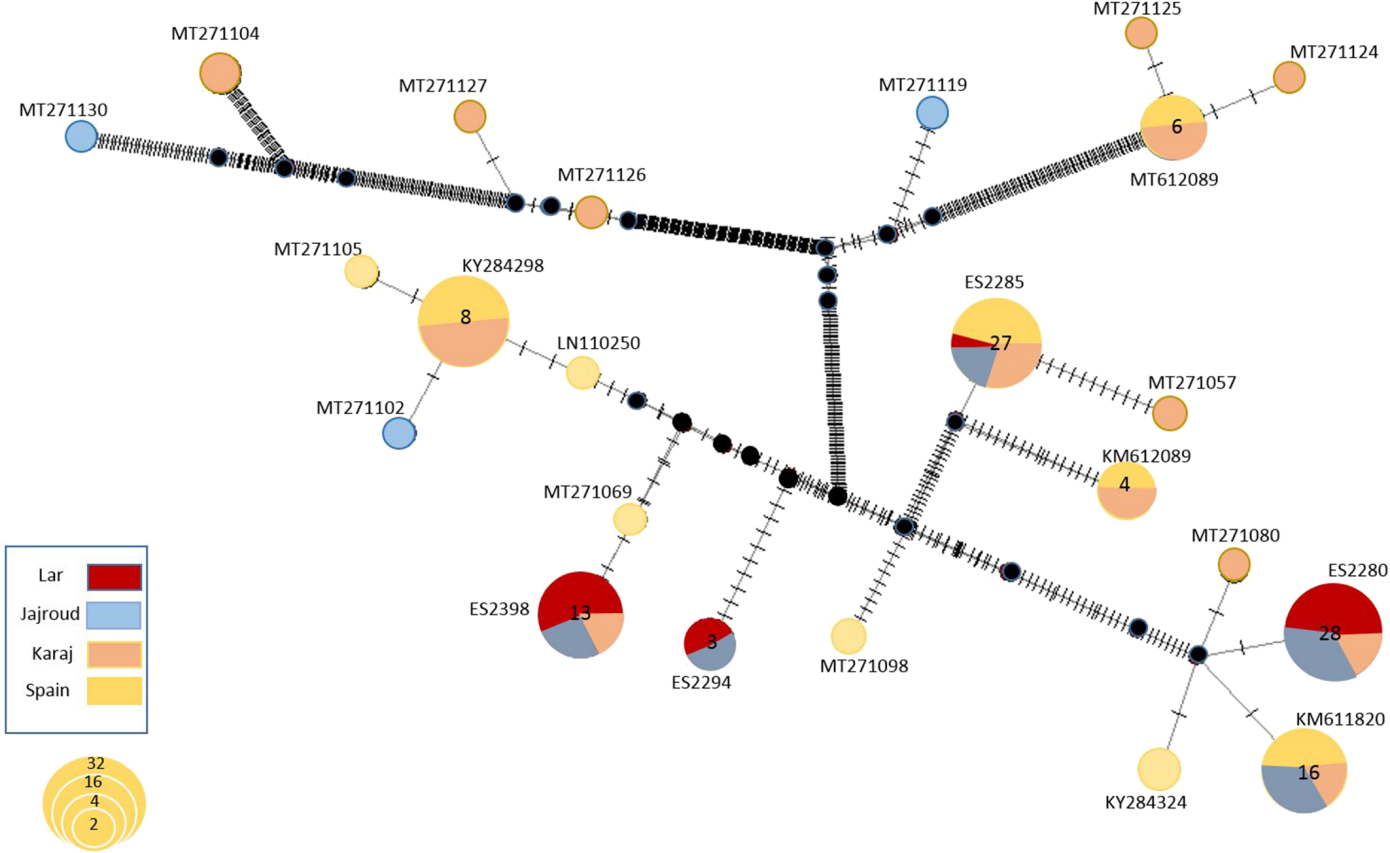
**Median-joining (MJ) haplotype network.** Each circle represents a unique haplogroup, and its size reflects the number of individuals expressing that haplotype. Crosshatches indicate the number of nucleotide differences between haplotypes. The geographic locations of the haplotypes are indicated in the legend. Lar, Lar River; Jajroud, Jajroud River, Karaj, Karaj River; Spain.

**Fig. 4.**
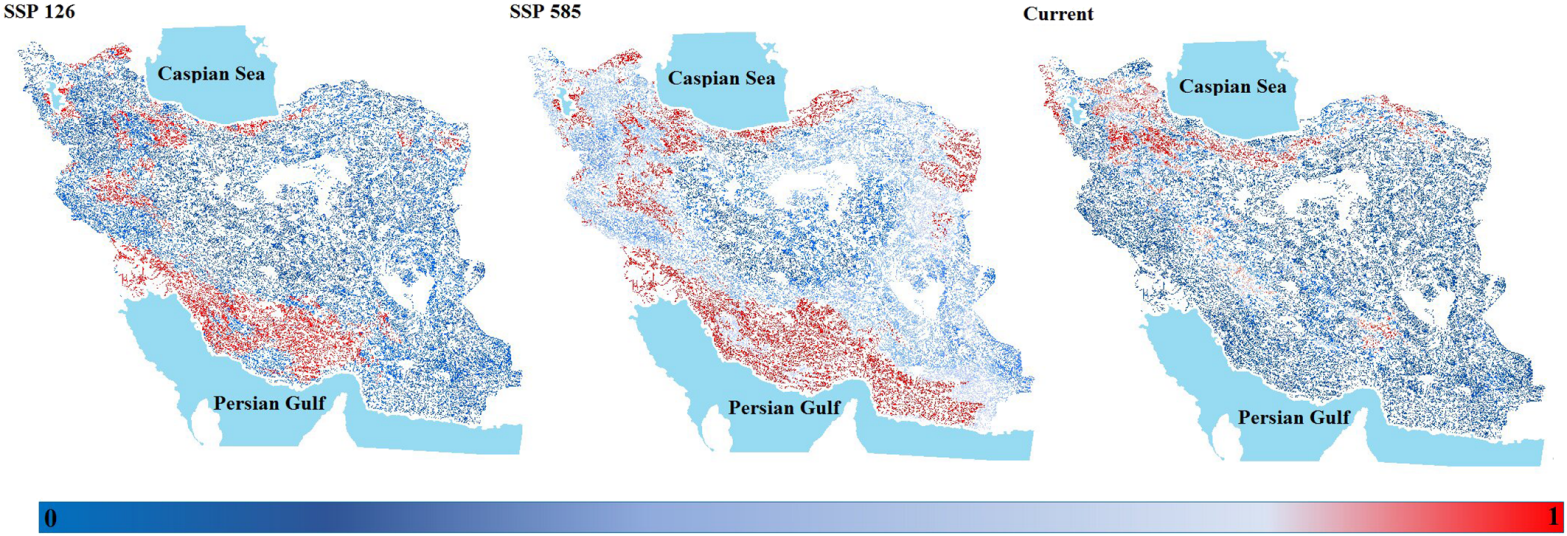
**Dynamic changes in the suitable habitat of *E. tetraedra* in the freshwaters of Iran under current and two future climate scenarios 2061–2080 (ssp 126 and ssp 585) based on MRI-CGCM3 model.** Maps are plotted with a high resolution (ca. 1 km) and natural-breaks classification, and a World Geodetic System projection (WGS 1984).

### DNA amplification and sequencing

The DNA was extracted from tissues using a standard high-salt method (Sambrook et al., 1989). The partial mitochondrial Cytochrome c Oxidase subunit I (COI) was amplified for all specimens using the universal primers forward LCO1490 (5′-TACTC-AACAA-ATCAC-AAAGA-TATTG-G-3′) and reverse HCO2198 (5′-TAAAC-TTCAGGGTGA-CCAAAAAATC-A-3′) (Shekhovtsov et al., 2016). Polymerase Chain Reactions (PCRs) were conducted with 1 μl template DNA (50–100 ng), 0.5 μl of each primer, 12.5 μl Master Mix Red (Ampliqon) and 10.5 μl of ddH2O up to 25 μl of reaction mix. The amplification of DNA was performed with an initial denaturation period of 3 min at 94°C followed by 34 cycles of 94°C for 30 s, primer-specific annealing temperature of 48°C for 30 s, 72°C for 1 min and a single final extension at 72°C for 5 min. The quality of PCR products was assessed with agarose gel 1% stained with Safe-Red™. The suitable amplicons were sent to Pishgam Inc., for purification and sequencing.

### Sequence analyses

Initially, the nucleotide sequences were edited by BioEdit v.2.34 (Hall, 1999), aligned by Geneious Prime^®^ v.2021.0.0 (Biomatters, www.geneious.com) and optimized by eye using MEGA X (Kumar et al., 2018). We extracted 117 sequences from Genbank which were added to our dataset (Table S1). The final gene dataset was 528 bp and MAFFT v.7 (https://mafft.cbrc.jp/alignment/server/) was used for alignment.

### Phylogenetic analyses and haplotype network

The datasets, with 528 bp length, included partial sequences of COI to investigate the position of individuals belonging to the *E. tetraedra* for a phylogeny tree was used. The tree based on 140 sequences examined in this research contains the Lar National Park (23 sequences), the Jajroud (40 sequences), and the Karaj River (40 sequences), and 37 sequences from the native distribution area of the species were used.

Phylogenetic analyses of *E. tetraedra* of the COI data were reconstructed using maximum-likelihood (ML) and Bayesian inference (BI) approaches. *Hermodice carunculata* downloaded from GenBank was used as an outgroup. The Akaike information criterion was used to select Nucleotide substitution models (GTR+I+G) in MrModeltest v.2 (Nylander, 2004).

We used IQ-Tree v.1.6.12 (Nguyen et al., 2015) to perform Maximum Likelihood (ML) inference. The confidence of branch supports was assessed using the ultrafast Bootstrap (UFB) approach (Hoang et al., 2018), with 1000 pseudoreplicates. For BI analysis, two independent runs and four Markov chains (three heated chains and a single cold chain) using the best-fit models were performed in MrBayes v.3.1.2 (Huelsenbeck and Ronquist, 2001). Each run was conducted with Markov Chain Monte Carlo (MCMC) sampling for 6 million generations and parameters were saved every 100 iterations, which produced 6001 trees during the analysis. Finally, 10% of the trees were discarded as burn-in and the remaining trees were used to reconstruct the consensus tree. Tracer v.1.7 (Rambaut et al., 2009) was implemented to the performance of each run and evaluate convergence. To edit and visualize the phylogenetic tree, FigTree v.1.4.4 (http://tree.bio.ed.ac.uk/software/figtree/) was used.

Also, we used NETWORK v.10.2 (Bandelt et al., 1999) to construct a median-joining network for COI.

### Genetic diversity and demographic analysis

The number of haplotypes (H), number of polymorphic sites (s), haplotype diversity (h) and nucleotide diversity (π) of each population were extracted by DnaSP v5 (Librado and Rozas, 2009).

For demographic history analyses, we used the selective neutrality test of Fu's Fs statistics (Fu, 1997) and Tajima's D (Tajima, 1989) based on COI to find evidence of recent expansion for each lineage using Arlequin v.3.5 (Excoffier and Lischer, 2010). A Mismatch Distribution (MMD) analysis was separately performed for each population to estimate the frequency distribution of the pairwise nucleotide differences, assuming a sudden expansion with spatial parameters. The test was performed using Arlequin v.3.5.

#### Environmental Variables and Model Construction

##### Occurrence Data

To SDMs, a total of 127 *E. tetraedra* locations were compiled between 2019 and 2023 through multiple sources (a) direct field surveys (b) the Global Biodiversity Information Facility (GBIF) website, and (c) distribution recorded by published papers (Javidkar et al., 2020; Nahavandi et al., 2021).

We visually checked occurrence points with those collected from the literature review in ArcGIS and to reduce spatial autocorrelation in the occurrence points all of them filtered with inaccurate spatial information using the package “CoordinateCleaner” (Zizka et al., 2019) in the R.v.4.1.3. This process reduced our presence records to 98 points that were available for the habitat modeling approach.

##### Environmental Variables

We used 19 environmental variables that were used to affect the spatial range of a species (Hermes et al., 2018). All bioclimatic variables (Bio1-Bio19) were downloaded from the WorldClim-Global Climate Database (https://www.worldclim.org/) with a resolution of ∼1 km^2^ (30-arc second) for both current (1970–2000) and future (2061–2080) climatic scenarios. Slope data was extracted from the Digital Elevation Model (DEM, http://www.worldclim.org) as an additional geographical input to provide a measure of topographic heterogeneity. In addition, the Human Footprint Model offered by Sanderson et al. (2002) to evaluate quantifies anthropogenic effects on the *E. tetraedra* habitat. All layers with WGS1984 datum were projected onto a UTM grid and resampled resolution at 1 km^2^. A principal Component Analysis (PCA) was estimated for multicollinearity among predictors by calculating coefficients (r<0.75) and criteria to select which essential variables in the distribution models for the present study.

Eventually, the remaining input variables for the modeling were as follows: annual mean temperature (Bio 1), temperature annual range (Bio 7), mean temperature of wettest quarter (Bio 8), mean temperature of driest quarter (Bio 9), precipitation of wettest month (Bio 13), precipitation of driest month (Bio 14), precipitation of wettest quarter (Bio 16), human footprint and slope.

For future mapping of the suitable climate of *E. tetradra* under future climate change, we extracted the bioclimatic data from MRI-CGCM3 (Meteorological Research Institute, Japan) and used two scenarios Shared Socio-economic Pathways (SSPs): 126 and 585 based on CMIP6. An ensemble model approach was applied to *E. tetraedra* distribution model (Thuiller et al., 2009) using the BIOMOD2 package (Thuiller et al., 2016) in R.v.4.1.3 (R Development Core Team, 2014). The ensemble model (Poulos et al., 2012) was formed using nine modeling techniques: generalized boosting method (GBM), the generalized linear model (GLM), maximum entropy (MaxEnt), flexible discriminant analysis (FDA), random forest (RF), classification tree analysis (CTA), multivariate adaptive regression splines (MARS), surface range envelops (SRE) and artificial neural network (ANN). To provide more accurate predictions we created many pseudo-absences (*n*=220 points) with five replicates per model (Hamid et al., 2019; Dar et al., 2021).

We also evaluated model performance using the Area Under the receiver operating Curve (AUC=ROC), the true skill statistic (TSS), and Cohen's Kappa (KAPPA) metrics.

## Supplementary Material

10.1242/biolopen.060180_sup1 Supplementary information
