# Supplementary material for: The threat of a non-native oligochaete species in Iran's freshwater: assessment of the diversity and origin of Eiseniella tetraedra (Savigny, 1826) and its response to climate change
Source: Biol Open. 2023 Dec 29;12(12):bio060180. doi: 10.1242/bio.060180 (PMC10840848; doi:10.1242/bio.060180)
Supplement: Supplementary information [file biolopen-12-060180-s1.pdf]

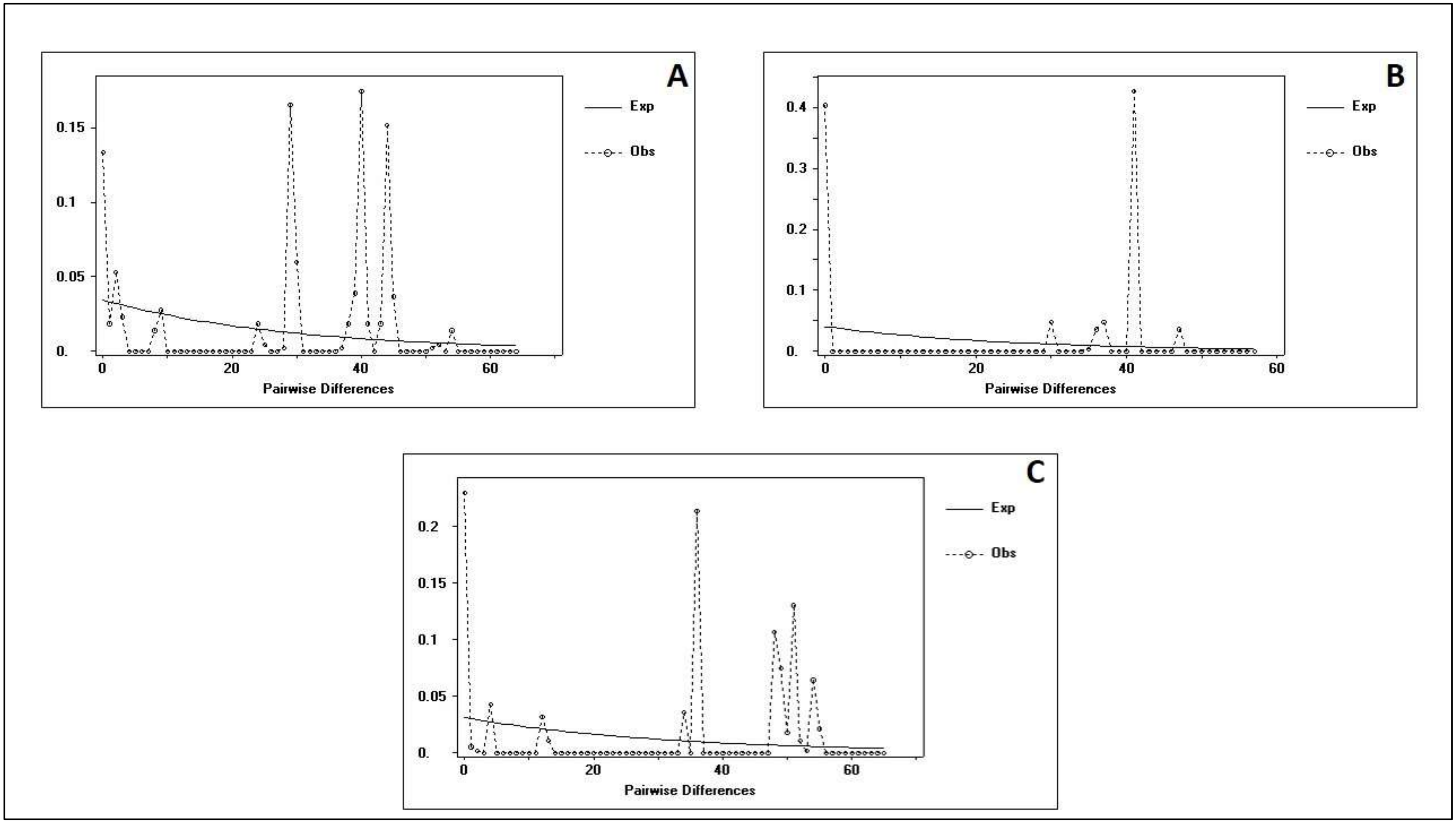

Fig. S1. Mismatch distributions of pairwise differences for A: Karaj River, B: Jajrud River, and C: Lar River. Depicted are observed (dotted line) and expected (black lines) frequencies obtained under a model allowing for demographic expansion.

Biology Open • Supplementary information

Table S1. List of species, Locality and GenBank accession numbers separated according to applied genes.

Species	Family	Ac number	Locality	Coordinates	GenBank Accession number
Eiseniella tetraedra	Lumbricidae	ES2302	Lar River: Delichay	35°55'03.6"N 51°59'32.4"E	OR798483
Eiseniella tetraedra	Lumbricidae	ES2303	Lar River: Delichay	35°58'12.7"N 52°00'02.4"E	OR798484
Eiseniella tetraedra	Lumbricidae	ES2280	Lar River: Delichay	35°58'08.9"N 51°59'37.8"E	OR798466
Eiseniella tetraedra	Lumbricidae	ES2281	Lar River: Delichay	35°57'02.6"N 51°55'58.9"E	OR798467
Eiseniella tetraedra	Lumbricidae	ES2304	Lar River: Sefidab	35°57'02.6"N 51°55'58.9"E	OR798485
Eiseniella tetraedra	Lumbricidae	ES2283	Lar River: Sefidab	35°56'09.1"N 51°56'52.9"E	OR798468
Eiseniella tetraedra	Lumbricidae	ES2284	Lar River: Sefidab	35°57'02.6"N 51°55'58.9"E	OR798469
Eiseniella tetraedra	Lumbricidae	ES2285	Lar River: Alarm	35°55'15.0"N 51°52'21.2"E	OR798470
Eiseniella tetraedra	Lumbricidae	ES2286	Lar River: Alarm	35°55'15.0"N 51°52'21.2"E	OR798471
Eiseniella tetraedra	Lumbricidae	ES2287	Lar River: gozal darh	35°55'08.8"N 51°52'55.4"E	OR798472
Eiseniella tetraedra	Lumbricidae	ES2306	Lar River: Siya plus	35°55'08.8"N 51°52'55.4"E	OR798486
Eiseniella tetraedra	Lumbricidae	ES2289	Lar River: Siya plus	35°53'20.1"N 51°53'12.4"E	OR798473
Eiseniella tetraedra	Lumbricidae	ES2290	Lar River: Siya plus	35°54'04.9"N 51°53'10.0"E	OR798474
Eiseniella tetraedra	Lumbricidae	ES2291	Lar River: Siya plus	35°54'58.4"N 51°53'01.2"E	OR798475
Eiseniella tetraedra	Lumbricidae	ES2307	Lar River: Siya plus	35°54'58.4"N 51°53'01.2"E	OR798487
Eiseniella tetraedra	Lumbricidae	ES2293	Lar River: Lar	35°53'50.5"N 51°50'46.6"E	OR798476
Eiseniella tetraedra	Lumbricidae	ES2294	Lar River: Lar	35°53'45.9"N 51°50'22.0"E	OR798477
Eiseniella tetraedra	Lumbricidae	ES2295	Lar River: Lar	35°54'30.0"N 51°49'12.0"E	OR798478
Eiseniella tetraedra	Lumbricidae	ES2301	Lar River: Lar	35°54'25.2"N 51°49'42.5"E	OR798482
Eiseniella tetraedra	Lumbricidae	ES2297	Lar River: Lar	35°57'25.6"N 51°46'49.9"E	OR798479
Eiseniella tetraedra	Lumbricidae	ES2298	Lar River: Dobradar	35°58'29.3"N 51°46'12.7"E	OR798488
Eiseniella tetraedra	Lumbricidae	ES2299	Lar River: Lar	35°53'59.5"N 51°50'36.6"E	OR798480
Eiseniella tetraedra	Lumbricidae	ES2300	Lar River: Lar	35°54'45.9"N 51°50'33.0"E	OR798481
Eiseniella tetraedra	Lumbricidae	–	Jajroud River: Abnik (lower course)	35857.5730N, 051834.7630E	MT271084
Eiseniella tetraedra	Lumbricidae	–	Jajroud River: Abnik (lower course)	35857.5730N, 051834.7630E	MT271088
Eiseniella tetraedra	Lumbricidae	–	Jajroud River: Abnik (lower course)	35857.5730N, 051834.7630E	MT271089
Eiseniella tetraedra	Lumbricidae	–	Jajroud River: Abnik (lower course)	35857.5730N, 051834.7630E	MT271076
Eiseniella tetraedra	Lumbricidae	–	Jajroud River: Abnik (lower course)	35857.5730N, 051834.7630E	MT271086

Eiseniella tetraedra	Lumbricidae	–	Jajroud River: Abnik (lower course)	35857.5730N, 051834.7630E	MT271062
Eiseniella tetraedra	Lumbricidae	–	Jajroud River: Abnik (lower course)	35857.5730N, 051834.7630E	MT271061
Eiseniella tetraedra	Lumbricidae	–	Jajroud River: Shemshak	35855.6810N, 051831.5420E	MT271112
Eiseniella tetraedra	Lumbricidae	–	Jajroud River: Shemshak	35855.6810N, 051831.5420E	MT271099
Eiseniella tetraedra	Lumbricidae	–	Jajroud River: Shemshak	35855.6810N, 051831.5420E	MT271065
Eiseniella tetraedra	Lumbricidae	–	Jajroud River: Fasham	35855.6750N, 051831.5630E	MT271119
Eiseniella tetraedra	Lumbricidae	–	Jajroud River: Fasham	35855.6750N, 051831.5630E	MT271056
Eiseniella tetraedra	Lumbricidae	–	Jajroud River: Ahar	35856.0390N, 051827.8080E	MT271087
Eiseniella tetraedra	Lumbricidae	–	Jajroud River: Ahar	35856.0390N, 051827.8080E	MT271117
Eiseniella tetraedra	Lumbricidae	–	Jajroud River: Ahar	35856.0390N, 051827.8080E	MT271082
Eiseniella tetraedra	Lumbricidae	–	Jajroud River: Ahar	35856.0390N, 051827.8080E	MT271081
Eiseniella tetraedra	Lumbricidae	–	Jajroud River: Ahar	35856.0390N, 051827.8080E	MT271080
Eiseniella tetraedra	Lumbricidae	–	Jajroud River: Ahar	35856.0390N, 051827.8080E	MT271079
Eiseniella tetraedra	Lumbricidae	–	Jajroud River: Haji-Abad	35852.5110N, 051832.1630E	MT271114
Eiseniella tetraedra	Lumbricidae	–	Jajroud River: Haji-Abad	35852.5110N, 051832.1630E	MT271102
Eiseniella tetraedra	Lumbricidae	–	Jajroud River: Haji-Abad	35852.5110N, 051832.1630E	MT271113
Eiseniella tetraedra	Lumbricidae	–	Jajroud River: Haji-Abad	35852.5110N, 051832.1630E	MT271101
Eiseniella tetraedra	Lumbricidae	–	Jajroud River: Haji-Abad	35852.5110N, 051832.1630E	MT271100
Eiseniella tetraedra	Lumbricidae	–	Jajroud River: Abnik (upper course)	35859.1970N, 051837.6970E	MT271083
Eiseniella tetraedra	Lumbricidae	–	Jajroud River: Abnik (upper course)	35859.1970N, 051837.6970E	MT271078
Eiseniella tetraedra	Lumbricidae	–	Jajroud River: Abnik (upper course)	35859.1970N, 051837.6970E	MT271115
Eiseniella tetraedra	Lumbricidae	–	Jajroud River: Abnik (upper course)	35859.1970N, 051837.6970E	MT271077
Eiseniella tetraedra	Lumbricidae	–	Jajroud River: Abnik (upper course)	35859.1970N, 051837.6970E	MT271116
Eiseniella tetraedra	Lumbricidae	–	Jajroud River: Saeed-Abad	35843.6750N, 051841.8660E	MT271054
Eiseniella tetraedra	Lumbricidae	–	Jajroud River: Saeed-Abad	35843.6750N, 051841.8660E	MT271074
Eiseniella tetraedra	Lumbricidae	–	Jajroud River: Saeed-Abad	35843.6750N, 051841.8660E	MT271063
Eiseniella tetraedra	Lumbricidae	–	Jajroud River: Saeed-Abad	35843.6750N, 051841.8660E	MT271060
Eiseniella tetraedra	Lumbricidae	–	Jajroud River: Saeed-Abad	35843.6750N, 051841.8660E	MT271058
Eiseniella tetraedra	Lumbricidae	–	Jajroud River: Saeed-Abad	35843.6750N, 051841.8660E	MT271059
Eiseniella tetraedra	Lumbricidae	–	Jajroud River: Saeed-Abad	35843.6750N, 051841.8660E	MT271064
Eiseniella tetraedra	Naididae	–	Jajroud River: Saeed-Abad	35843.6750N, 051841.8660E	MT271128
Eiseniella tetraedra	Naididae	–	Jajroud River: Saeed-Abad	35843.6750N, 051841.8660E	MT271129
Eiseniella tetraedra	Lumbricidae	–	Jajroud River: Saeed-Abad	35843.6750N, 051841.8660E	MT271055

Biology Open • Supplementary information

Eiseniella tetraedra	Lumbricidae	–	Jajroud River: Saeed-Abad	35843.6750N, 051841.8660E	–
Eiseniella tetraedra	Naididae	–	Jajroud River: Saeed-Abad	35843.6750N, 051841.8660E	MT271130
Eiseniella tetraedra	Haplotaxidae	–	Karaj River: Shahrestanak	35857.7630N, 051821.9950E	–
Eiseniella tetraedra	Haplotaxidae	–	Karaj River: Shahrestanak	35857.7630N, 051821.9950E	–
Eiseniella tetraedra	Lumbriculidae	–	Karaj River: Shahrestanak	35857.7630N, 051821.9950E	MT271126
Eiseniella tetraedra	Haplotaxidae	–	Karaj River: Shahrestanak	35857.7630N, 051821.9950E	MT271123
Eiseniella tetraedra	Haplotaxidae	–	Karaj River: Shahrestanak	35857.7630N, 051821.9950E	MT271122
Eiseniella tetraedra	Lumbriculidae	–	Karaj River: Shahrestanak	35857.7630N, 051821.9950E	MT271127
Eiseniella tetraedra	Haplotaxidae	–	Karaj River: Shahrestanak	35857.7630N, 051821.9950E	MT271124
Eiseniella tetraedra	Haplotaxidae	–	Karaj River: Shahrestanak	35857.7630N, 051821.9950E	MT271125
Eiseniella tetraedra	Lumbricidae	–	Karaj River: Shahrestanak 2	35858.5880N, 051820.1150E	MT271069
Eiseniella tetraedra	Lumbricidae	–	Karaj River: Shahrestanak 2	35858.5880N, 051820.1150E	MT271085
Eiseniella tetraedra	Lumbricidae	–	Karaj River: Shahrestanak 2	35858.5880N, 051820.1150E	MT271075
Eiseniella tetraedra	Lumbricidae	–	Karaj River: Shahrestanak 2	35858.5880N, 051820.1150E	MT271068
Eiseniella tetraedra	Lumbricidae	–	Karaj River: Velayatroud	3684.230N, 51823.3260E	MT271098
Eiseniella tetraedra	Lumbricidae	–	Karaj River: Velayatroud	3684.230N, 51823.3260E	MT271073
Eiseniella tetraedra	Lumbricidae	–	Karaj River: Velayatroud 4	36805.8130N, 51821.4760E	MT271106
Eiseniella tetraedra	Lumbricidae	–	Karaj River: Velayatroud 4	36805.8130N, 51821.4760E	MT271096
Eiseniella tetraedra	Lumbricidae	–	Karaj River: Velayatroud 4	36805.8130N, 51821.4760E	MT271110
Eiseniella tetraedra	Lumbricidae	–	Karaj River: Velayatroud 4	36805.8130N, 51821.4760E	MT271092
Eiseniella tetraedra	Lumbricidae	–	Karaj River: Velayatroud 4	36805.8130N, 51821.4760E	MT271111
Eiseniella tetraedra	Lumbricidae	–	Karaj River: Nojan	35855.6350N, 5183.5450E	MT271057
Eiseniella tetraedra		–	Karaj River: Nojan	35855.6350N, 5183.5450E	MT271120
Eiseniella tetraedra	Lumbricidae	–	Karaj River: Nojan	35855.6350N, 5183.5450E	MT271071
Eiseniella tetraedra	Lumbricidae	–	Karaj River: Nojan	35855.6350N, 5183.5450E	MT271072
Eiseniella tetraedra		–	Karaj River: Nojan	35855.6350N, 5183.5450E	MT271121
Eiseniella tetraedra	Lumbricidae	–	Karaj River: Nojan	35855.6350N, 5183.5450E	MT271070
Eiseniella tetraedra	Lumbricidae	–	Karaj River: Doab	36806.7850N, 51819.6850E	MT271095
Eiseniella tetraedra	Lumbricidae	–	Karaj River: Doab	36806.7850N, 51819.6850E	MT271097
Eiseniella tetraedra	Lumbricidae	–	Karaj River: Doab	36806.7850N, 51819.6850E	MT271108
Eiseniella tetraedra	Lumbricidae	–	Karaj River: Doab	36806.7850N, 51819.6850E	MT271109
Eiseniella tetraedra	Lumbricidae	–	Karaj River: Doab	36806.7850N, 51819.6850E	MT271091
Eiseniella tetraedra	Lumbricidae	–	Karaj River: Doab	36806.7850N, 51819.6850E	MT271103
Eiseniella tetraedra	Lumbricidae	–	Karaj River: Nojan	35855.3810N, 51803.0960E	MT271066
Eiseniella tetraedra	Lumbricidae	–	Karaj River: Nojan	35855.3810N, 51803.0960E	MT271105
Eiseniella tetraedra	Lumbricidae	–	Karaj River: Nojan	35855.3810N, 51803.0960E	MT271067

Biology Open • Supplementary information

Eiseniella tetraedra	Lumbricidae	–	Karaj River: Nojan	35855.3810N, 51803.0960E	MT271090
Eiseniella tetraedra	Lumbricidae	–	Karaj River: Nojan	35855.3810N, 51803.0960E	MT271104
Eiseniella tetraedra	Lumbricidae	–	Karaj River: Nojan	35855.3810N, 51803.0960E	MT271094
Eiseniella tetraedra	Lumbricidae	–	Karaj River: Polekhab	3680.830N, 51808.6030E	MT271093
Eiseniella tetraedra	Lumbricidae	–	Karaj River: Polekhab	3680.830N, 51808.6030E	MT271118
Eiseniella tetraedra	Lumbricidae	–	Karaj River: Polekhab	3680.830N, 51808.6030E	MT271107
Eiseniella tetraedra	Lumbricidae	–	Spain, Madrid	–	KY284226.1
Eiseniella tetraedra	Lumbricidae	–	Switzerland	–	LT903805.1
Eiseniella tetraedra	Lumbricidae	–	Switzerland, Geneva	–	LN810250.1
Eiseniella tetraedra	Lumbricidae	–	Spain, Madrid	–	KY284297.1
Eiseniella tetraedra	Lumbricidae	–	Spain, Madrid	–	KY284298.1
Eiseniella tetraedra	Lumbricidae	–	Spain, Madrid	–	KY284296.1
Eiseniella tetraedra	Lumbricidae	–	Spain, Madrid	–	KY284302.1
Eiseniella tetraedra	Lumbricidae	–	France, Ardeche	–	MF458696.1
Eiseniella tetraedra	Lumbricidae	–	Canada, Alberta	–	KM612089.1
Eiseniella tetraedra	Lumbricidae	–	Canada, Alberta	–	KM612254.1
Eiseniella tetraedra	Lumbricidae	–	Spain, Galicia	–	KY284261.1
Eiseniella tetraedra	Lumbricidae	–	Spain, Madrid	–	KY284221.1
Eiseniella tetraedra	Lumbricidae	–	Spain, Madrid	–	KY284189.1
Eiseniella tetraedra	Lumbricidae	–	Spain, Galicia	–	KY284292.1
Eiseniella tetraedra	Lumbricidae	–	Spain, Galicia	–	KY284288.1
Eiseniella tetraedra	Lumbricidae	–	Spain, Galicia	–	KY284287.1
Eiseniella tetraedra	Lumbricidae	–	Spain, Galicia	–	KY284286.1
Eiseniella tetraedra	Lumbricidae	–	Spain, Galicia	–	KY284281.1
Eiseniella tetraedra	Lumbricidae	–	Spain, Galicia	–	KY284257.1
Eiseniella tetraedra	Lumbricidae	–	Spain, Madrid	–	KY284191.1
Eiseniella tetraedra	Lumbricidae	–	Spain, Madrid	–	KY284330.1
Eiseniella tetraedra	Lumbricidae	–	Spain, Madrid	–	KY284324.1
Eiseniella tetraedra	Lumbricidae	–	Canada, British Columbia	–	KM611822.1
Eiseniella tetraedra	Lumbricidae	–	Australia, Western Australia	–	KT073958.1
Eiseniella tetraedra	Lumbricidae	–	Spain, Madrid	–	KY284329.1
Eiseniella tetraedra	Lumbricidae	–	Spain, Galicia	–	KY284326.1
Eiseniella tetraedra	Lumbricidae	–	Spain, Galicia	–	KY284309.1
Eiseniella tetraedra	Lumbricidae	–	Spain, Galicia	–	KY284310.1
Eiseniella tetraedra	Lumbricidae	–	Spain, Galicia	–	KY284311.1
Eiseniella tetraedra	Lumbricidae	–	Spain, Galicia	–	KY284308.1
Eiseniella tetraedra	Lumbricidae	–	Spain, Galicia	–	KY284307.1
Eiseniella tetraedra	Lumbricidae	–	Spain, Galicia	–	KY284306.1
Eiseniella tetraedra	Lumbricidae	–	Spain, Madrid	–	KY284223.1
Eiseniella tetraedra	Lumbricidae	–	USA, New York	–	MK558022.1
Eiseniella tetraedra	Lumbricidae	–	USA, New York	–	MK505453.1
Eiseniella tetraedra	Lumbricidae	–	USA, New York	–	MK505451.1
Eiseniella tetraedra	Lumbricidae	–	USA	–	HQ983627.1
Hermodice carunculata	Amphinomidae	–	Greece	–	KF878476.1

Table S2. Evaluation of nine applied models predicting *E. tetraedra* distribution in Iran freshwater using AUC, TSS and KAPPA.

	SRE	MARS	FDA	GLM	MaxEnt	ANN	CTA	GBM	RF
**AUC**	0.98	0.91	0.97	1	0.99	1	0.95	0.96	0.98
**TSS**	0.74	0.71	0.74	0.88	0.86	0.89	0.71	0.76	0.88
**KAPPA**	0.82	0.77	.079	0. 89	0.94	0.82	0.88	.089	.091
